# Supplementary figures and images for: Integrating Polygenic Scores into Multifactorial Breast Cancer Risk Assessment: Insights from the First Year of Clinical Implementation in Western Austria
Source: Cancers (Basel). 2025 Jul 26;17(15):2472. doi: 10.3390/cancers17152472 (PMC12346286; doi:10.3390/cancers17152472)

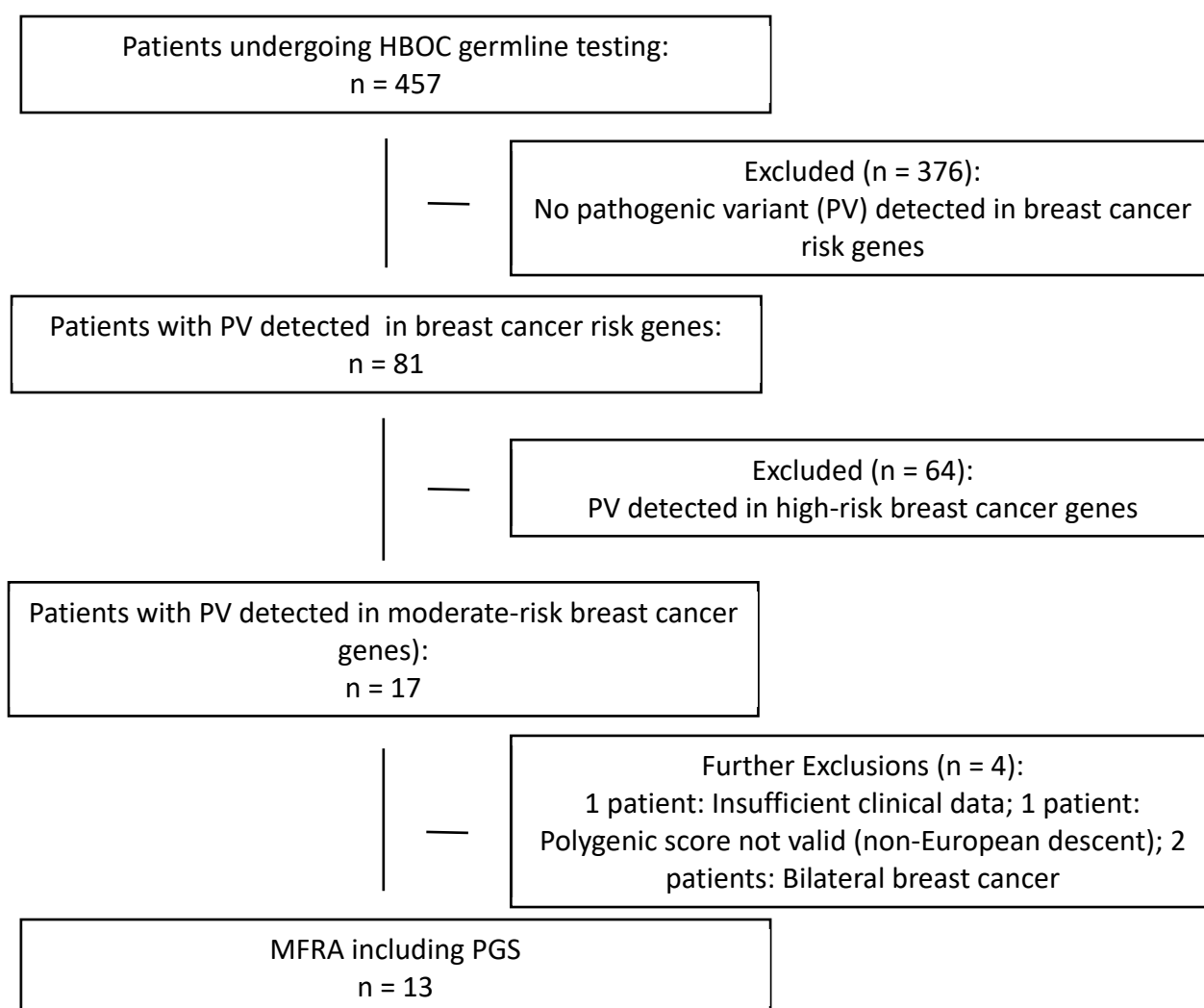

**Supplementary Figure S1.** CONSORT diagram depicting the ex- and inclusion of patients.

Supplement: Supplementary file 1 [file cancers-17-02472-s001.zip › cancers-3702756-supplementary.pdf]
